# Scintillation Response of Nd-Doped LaMgAl_11_O_19_ Single Crystals Emitting NIR Photons for High-Dose Monitoring

**DOI:** 10.3390/s22249818

**Published:** 2022-12-14

**Authors:** Daisuke Nakauchi, Takumi Kato, Noriaki Kawaguchi, Takayuki Yanagida

**Affiliations:** Division of Materials Science, Nara Institute of Science and Technology (NAIST), 8916-5 Takayama, Ikoma 630-0192, Nara, Japan

**Keywords:** scintillator, scintillation detector radiation measurement, dosimetry, photoluminescence, radioluminescence, afterglow

## Abstract

The Nd-doped LaMgAl_11_O_19_ single crystals were synthesized by the floating zone method, and the photoluminescence and scintillation properties were evaluated. Under X-ray irradiation, several sharp emission peaks due to the 4f–4f transitions of Nd^3+^ were observed at 900, 1060, and 1340 nm in the near-infrared range, and the decay curves show the typical decay time for Nd^3+^. The samples show good afterglow properties comparable with practical X-ray scintillators. The 1% and 3% Nd-doped LaMgAl_11_O_19_ samples show a good linearity in the dynamic range from 6–60,000 mGy/h.

## 1. Introduction

A scintillator is a phosphor that emits photons with several eV when irradiated by ionizing radiations and has been used in applications such as medical diagnostic [1,2,3], security [4,5,6,7], and resource exploration, etc. [8,9,10,11,12]. A scintillation detector consists of a scintillator that converts ionizing radiations into photons and a photodetector such as a photomultiplier tube or photodiode that converts photons into electrical signals. Since the conversion efficiency from photon to electrical signal depends on the quantum efficiency of the photodetector, phosphors that emit in the UV–visible region, where the above photodetectors have high sensitivity, have been investigated [13,14,15,16].

Since the nuclear accident in Fukushima, various countermeasures using materials have been studied for reconstruction [17,18,19,20]. In recent years, scintillators which exhibit luminescence in the near-infrared (NIR) region have attracted attention for remote monitoring applications in high-dose fields [21,22,23]. Remote monitoring using scintillators and optical fibers has been proposed to avoid radiation damage to semiconductors and electrical circuits of signal amplifiers involved in measurements [21,24,25,26,27,28]. Since the method uses a long optical fiber, the transmission efficiency of scintillation photons in an optical fiber is important. In the NIR region of 800–1700 nm, the transmittance of a quartz fiber is higher than that in the UV–visible region [29,30,31,32,33], and the transmission loss can be suppressed. So far, there have been sporadic studies on NIR-scintillators. In past studies, only emission wavelengths shorter than 1000 nm were monitored because a Si-photodiode was used as the photodetector. On the other hand, InGaAs-based photodiodes enable stable measurement of NIR scintillation photons with wavelengths longer than 1000 nm. In our previous works, various scintillators doped with Nd as an emission center in the NIR region have been developed as candidate materials [34,35,36,37,38].

LaMgAl_11_O_19_ has been extensively studied in various phosphor fields such as laser [39,40,41,42] and white LED applications [43,44,45,46,47,48]. Since LaMgAl_11_O_19_ has La^3+^ sites that can be easily substituted with doped rare-earth ions as an emission center, LaMgAl_11_O_19_ doped with various trivalent rare-earth ions such as Ce, Nd, Sm, Eu, Dy, Tm, an Yb, etc. [49,50,51,52,53,54,55] has been investigated. In addition, compared with conventional oxide scintillators such as rare-earth-based silicate and aluminate compounds [56,57,58,59,60,61,62,63,64], the composition ratio of rare-earth elements is relatively low, and the main component is Al_2_O_3_, so the cost of raw materials can be suppressed. However, no research on the scintillation properties for measuring ionizing radiations has been reported so far. In this study, the Nd-doped LaMgAl_11_O_19_ single crystals were synthesized, and the photoluminescence (PL) and scintillation properties were investigated to evaluate the potential applicability for high-dose monitoring applications.

## 2. Materials and Methods

To begin, 0.1, 0.3, 1, and 3 atomic% Nd-doped LaMgAl_11_O_19_ crystals were synthesized by the floating zone method. La_2_O_3_ (99.99%), MgO (99.99%), Al_2_O_3_ (99.99%), and Nd_2_O_3_ (99.99%) were used as raw materials. At first, mixed powders were molded into cylindrical shapes by using hydrostatic pressure and then sintered at 1400 °C for 8 h. After that, the prepared sample was used to grow a single crystal by using a floating zone furnace (FZD0192, Canon Machinery, Kusats, Japan). During the crystal growth, the growth speed was set to 5 mm/h, and the rotation speed was 20 rpm. To demonstrate the crystalline phase of the target compound, powder X-ray diffraction (XRD) patterns were obtained by using a diffractometer (MiniFlex600, Rigaku, Tokyo, Japan).

PL contour spectra and quantum yield (QY) were measured using Quantaurus-QY Plus (C13534, Hamamatsu Photonics, Shizuoka, Japan). The QY was calculated by monitoring emission wavelengths from 800 to 1700 nm under an excitation wavelength of 590 nm with a band width of 10 nm. PL decay curves were measured using Quantaurus-τ (C11367, Hamamatsu Photonics).

Scintillation properties, emission spectra, decay curves, and afterglow under X-rays were measured using laboratory-made setup [65,66]. To evaluate dose rate response, signal intensities were measured under X-rays with various irradiation dose rates by using a laboratory-made setup based on an InGaAs-PIN photodiode (G12180-250A, Hamamatsu Photonics) [35].

## 3. Results

**Figure 1:**
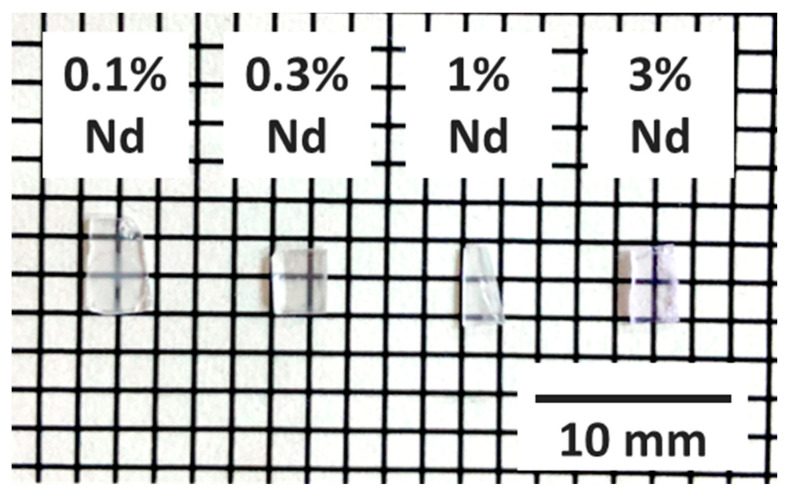
A photograph of the prepared LaMgAl_11_O_19_:Nd. The grid shows 2 mm intervals per division.

The synthesized samples were cut into small size, and the large surfaces were polished to the thickness of ~0.5 mm for the following characterizations. The photograph of the Nd-doped LaMgAl_11_O_19_ samples is shown in Figure 1. They are visibly transparent, and the 3% Nd-doped sample appears pale purple, while the rests appear colorless. The coloring is due to typical absorption of Nd^3+^.

**Figure 2:**
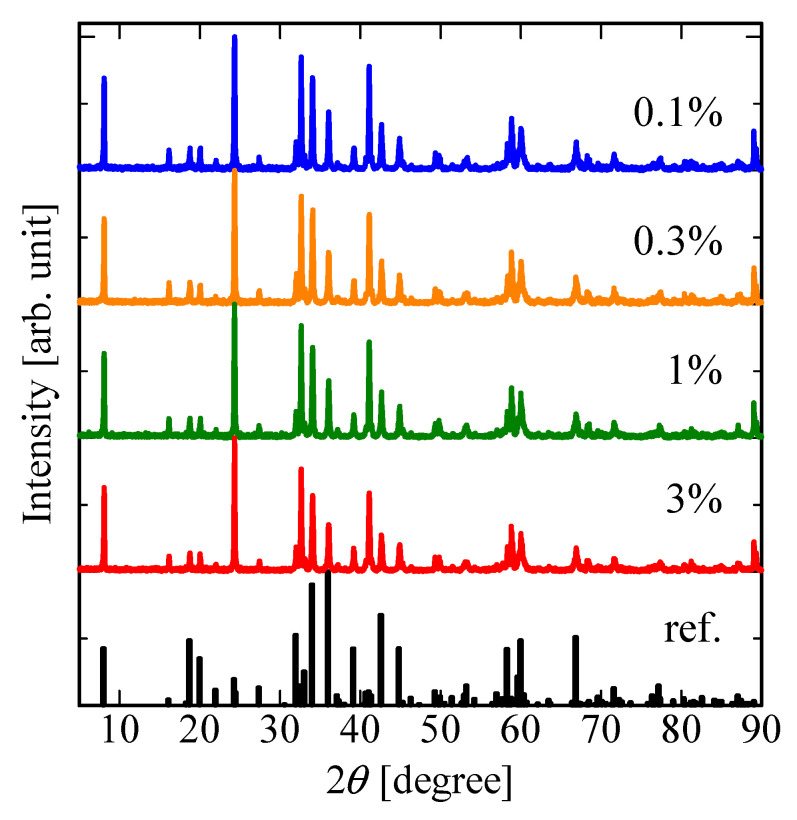
XRD patterns of LaMgAl_11_O_19_:Nd.

The powder XRD patterns of the LaMgAl_11_O_19_:Nd crystalline powders are illustrated in Figure 2, and the reference pattern (Crystal Open Database: 2002336) is also shown. The XRD peaks are in good agreement with the reference peaks, and no other peaks ascribed to the impurity phase can be observed. From the patterns, the samples have a single phase of LaMgAl_11_O_19_ with a hexagonal symmetry (space group: *P*6_3_/*mmc*). No significant peak-shifts owing to Nd-concentration are observed because the ionic radii of La^3+^ and Nd^3+^ is close.

**Figure 3:**
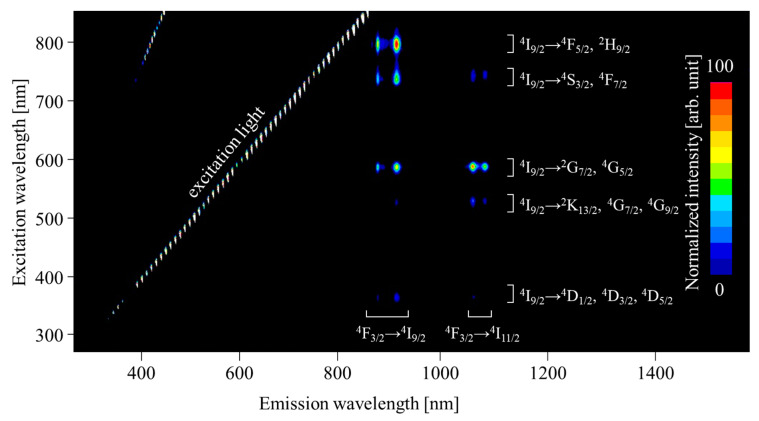
A PL contour spectrum of 1% Nd-doped LaMgAl_11_O_19_. Red and blue indicate high and low emission intensity, respectively.

In PL evaluation, all the LaMgAl_11_O_19_:Nd samples show similar spectral features, and the PL contour spectrum of the 1% Nd-doped samples are shown in Figure 3 as a representative. Two double peaks are observed at ~900 and 1060 nm, and the emission origins are ^4^F_3/2_–^4^I_9/2_ and ^4^F_3/2_–^4^I_11/2_, respectively [67]. All the samples show the highest QY under excitation at 580 nm, and the 0.1, 0.3, and 1% Nd-doped LaMgAl_11_O_19_ exhibit QY of 91.0%, 73.8%, 72.1%, and 70.9%, respectively. The 0.1% Nd-doped LaMgAl_11_O_19_ shows the highest value among the present samples, and QY monotonically decreases with Nd-concentration because of concentration quenching.

**Figure 4:**
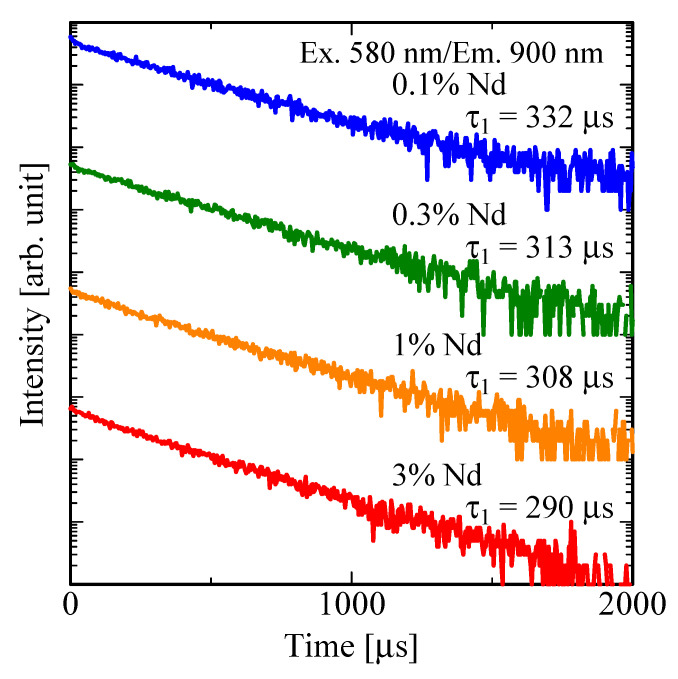
PL decay curves of LaMgAl_11_O_19_:Nd monitored at 900 nm when excited at 580 nm.

Figure 4 illustrates PL decay curves of Nd-doped LaMgAl_11_O_19_ monitored at ~900 nm under excitation at ~580 nm. The decay curves were fitted by the least squares method and consist of one exponential decay function. The obtained decay time constants (*τ*) were 332 μs for 0.1%, 313 μs for 0.3%, 308 μs for 1%, and 290 μs for 3%, respectively. They were almost the same value as the reported decay constant of Nd-doped LaMgAl_11_O_19_ (~300 μs [67]) and typical for the 4f–4f transitions of Nd^3+^ [68]. The decay time monotonically decreases with Nd-concentration. On the basis on the obtained results of *QY* and *τ*, the radiative (*k*_f_) and nonradiative (*k*_nr_) decay rates were calculated by using Equations (1) and (2).

*k*_f_ = *QY*/*τ*, (1)

*k*_nr_ = (1 − *QY*)/*τ*, (2)

The calculated values are listed in Table 1. *k*_f_ values are almost the same in all the samples, while *k*_nr_ monotonically increases with the dopant concentration. This trend indicates typical concentration quenching.

**Figure 5:**
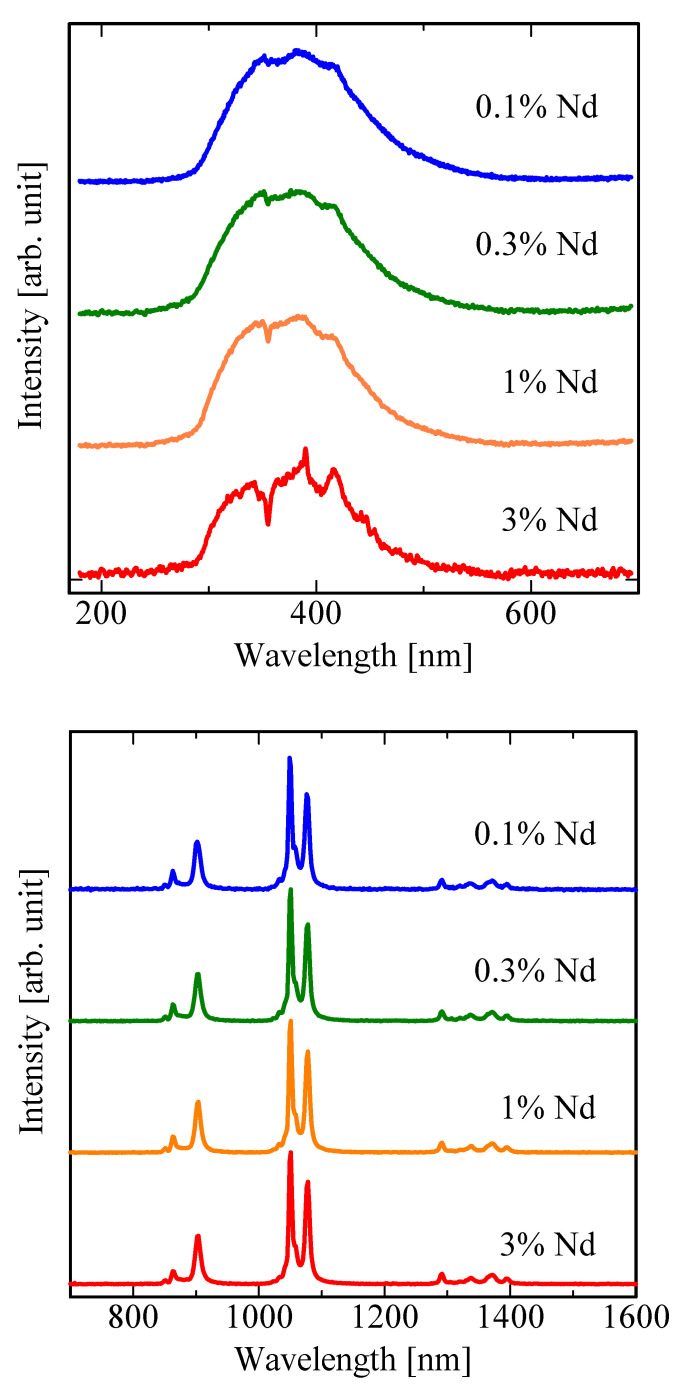
X-ray-induced emission spectra of LaMgAl_11_O_19_:Nd in the UV–visible (**top**) and NIR range (**bottom**).

Figure 5 show the X-ray-induced emission spectra in the range of UV–visible and NIR regions. A broad emission peak, possibly due to intrinsic luminescence of the host, and an absorption signal due to the 4f–4f (^4^I_9/2_–^4^D_1/2_, ^4^D_3/2_, ^4^D_5/2_) transitions of Nd^3+^ are also observed at 350 nm [69,70,71,72]. In addition, two sharp emission peaks appear at 390 and 410 nm, which are attributable to ^2^F_5/2_–^4^F_3/2_ and ^2^F_5/2_–^4^F_5/2_ transitions of Nd^3+^ [73]. These absorption and emission signals observed at 350–410 nm are the most clearly observed in the 3% Nd–doped sample. Investigation into the intrinsic luminescence is beyond the scope of this study. The spectral shape in the NIR region is almost the same as the PL spectra. Three groups of a few sharp emission peaks are observed at ~900, ~1060 and ~1340 nm, which are ascribed to ^4^F_3/2_–^4^I_9/2_, ^4^F_3/2_–^4^I_11/2_, and ^4^F_3/2_–^4^I_13/2_ transitions of Nd^3+^.

**Figure 6:**
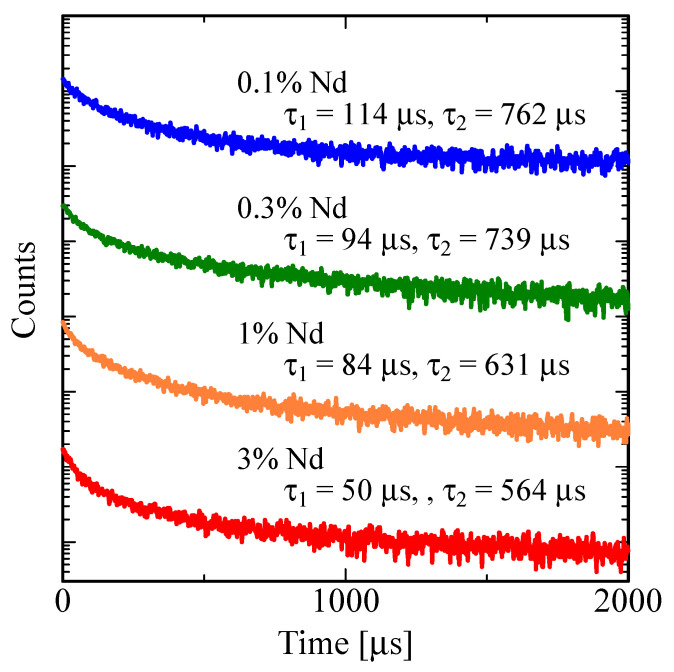
Decay curves of LaMgAl_11_O_19_:Nd under X-ray irradiation. The sensitivity of the used photomultiplier tube covers 380–920 nm.

Figure 6 shows the decay curves under X-ray irradiation. All the decay curves are approximated by a sum of two exponential decay functions. As summarized in Table 2, both the first (τ_1_) and second (τ_2_) decay time constants decrease as the dopant concentration increases. Since the obtained decay time constants are close to those in the previous reports on PL decay [74], they are due to emission at ~400 nm (^2^F_5/2_–^4^F_3/2_ and ^2^F_5/2_–^4^F_5/2_) and ~900 nm (^4^F_3/2_–^4^I_9/2_), respectively. The decay time (500–800 μs) corresponding to the emission at ~900 nm is longer than PL decay time (~300 μs) in Figure 3. PL occurs only by electronic transitions at an emission center, while X-ray-induced emission involves an additional energy transport process from a host to an emission center. Therefore, X-ray-induced decay time is longer than PL in most materials. The obtained decay constants are relatively long in comparison with conventional scintillators for the photon-counting applications. However, the decay time is acceptable because the integrated current is read out every few milliseconds in the monitoring applications.

**Figure 7:**
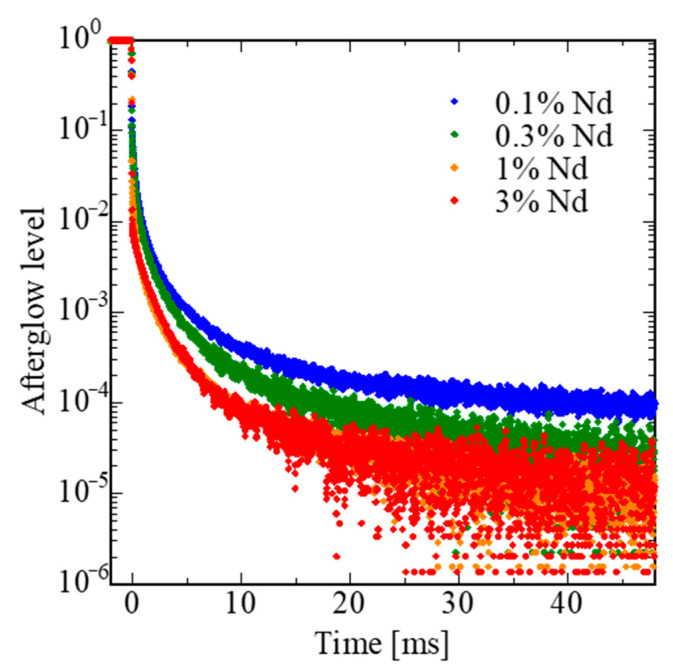
Afterglow curves of LaMgAl_11_O_19_:Nd after 2 ms X-ray irradiation.

Figure 7 shows the afterglow curves after X-ray irradiation for 2 ms. The afterglow levels at 20 ms (*AG*) were calculated by following Equation (3):

*AG* = (*I*_2_ − *I*_BG_)/(*I*_1_ − *I*_BG_), (3)

where *I*_BG_, *I*_1_, and *I*_2_ were signal intensity before, during, and after X-ray irradiation, respectively. The afterglow at *t* = 20 ms was 131 ppm for 0.1%, 62 ppm for 0.3%, 38 ppm for 1%, and 21 ppm for 3%. The obtained values were comparable with practical scintillators with low afterglow such as BGO (~10 ppm [75]). The results demonstrate suitability for monitoring applications.

**Figure 8:**
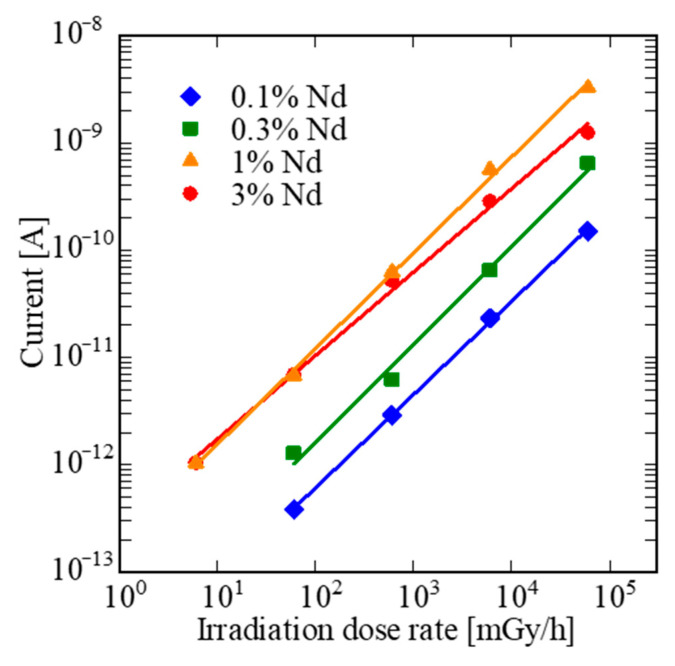
Relationships between dose rate and signal intensity of LaMgAl_11_O_19_:Nd.

Figure 8 shows the relationships between X-ray irradiation dose rate and the output signal. The evaluation simply simulates actual measurements in a high-dose field. The tested dose rates were from 6 to 60,000 mGy/h. The 1% and 3% Nd-doped samples show the lowest measurable dose rate of 6 mGy/h among the samples, and the measurable limits of the 0.1% and 0.3% Nd-doped LaMgAl_11_O_19_ sample were 60 mGy/h. Considering the afterglow characteristic results, 3% Nd-doped LaMgAl_11_O_19_ is the most suitable for detector uses among the samples. Compared with the past study using scintillator-emitting visible photons, the detection limit is superior to that of Pr:Gd_2_O_2_S coupled with Si-photodiode (0.8 Gy/h) [21] and comparable with those of some Nd-doped scintillators in our previous works such as GdVO_4_:Nd (6 mGy/h [35]), Bi_4_Ge_3_O_12_:Nd (10 mGy/h [36]), and SrY_2_O_4_:Nd (60 mGy/h [34]). Among them, the ratio of expensive raw materials is relatively small in LaMgAl_11_O_19_, which is advantageous from the viewpoint of the reasonable production cost.

## 4. Conclusions

Nd-doped LaMgAl_11_O_19_ single crystals were prepared by the FZ method, and the PL and scintillation properties were investigated. Under X-ray irradiation, several sharp emission peaks due to 4f–4f transitions of Nd^3+^ were clearly observed in the NIR range. The afterglow levels after X-ray irradiation for 2 ms improve as the Nd-concentration increases. The afterglow levels are comparable to practical X-ray scintillators with low afterglow such as Bi_4_Ge_3_O_12_. From the evaluation of relationships between X-ray irradiation dose rate and output signal, all the samples show a good linearity in the wide dynamic range. The 1% and 3% Nd-doped samples show the lowest detection limit of 6 mGy/h among the samples. Overall, the scintillation output and afterglow properties indicate that the 3% Nd-doped sample is the best among the prepared samples, and Nd-doped LaMgAl_11_O_19_ has a potential to be applied to scintillation detectors for monitoring high-dose fields.

## Figures and Tables

**Table 1:** PL *QY*, decay time constant (*τ*), radiative (*k*_f_) and nonradiative decay rates (*k*_nr_) calculated from PL *QY* and decay time constant.

	PL *QY* [%]	*τ* [s]	*k*_f_ [s^−1^]	*k*_nr_ [s^−1^]
0.1% Nd	91.0	3.32 × 10^−4^	2.74 × 10^3^	2.71 × 10^2^
0.3% Nd	73.8	3.13 × 10^−4^	2.36 × 10^3^	8.37 × 10^2^
1% Nd	72.1	3.08 × 10^−4^	2.34 × 10^3^	9.06 × 10^2^
3% Nd	70.9	2.90 × 10^−4^	2.45 × 10^3^	1.00 × 10^3^

**Table 2:** Decay time constants of first (*τ*_1_) and second (*τ*_2_) components calculated from the decay curves under X-ray irradiation.

	*τ*_1_ [μs]	*τ*_2_ [μs]
0.1% Nd	114	762
0.3% Nd	94	739
1% Nd	84	631
3% Nd	50	564

## Data Availability

Not applicable.
